# The Medical Work of the Local Government Board

**Published:** 1893-11-25

**Authors:** 


### The Medical Work of the Local Government Board.

### V.—DIPHTHERIA AND INFECTIVE ENDOCARDITIS.

There does not seem to be, on the face of things, any likeness between diphtheria and endocarditis. The one is, to the popular mind, heart disease, the other throat trouble, Why, then, do they stand side by side in Dr. Sidney Martin's contribution to Dr. Thorne Thorne's report? Because in both the action of a primary infective agent proceeds in a similar fashion. In the first place there is a mechanical action. In diphtheria there is the formation of a false membrane which covers the larynx and produces suffocation. It is to undo the effect of this obstruction of the air passages that tracheotomy is resorted to; and that the operation is often useless to save life is due to other and non-mechanical causes. In endocarditis a similar mechanical action takes place. Fungative vegetations, akin to that of the diphtheritic membrane, interfere with the action of the valves of the heart, and emboli, broken off from them, plug large blood-vessels, and prevent the supply of blood to the heart, as the diphtheritic membrane prevents the supply of air to the lungs. But these mechanical effects may be regarded as accidents, pathological details which are important enough in their bearing on life, and cannot be disregarded in treatment; but are not to be looked upon as the primary cause of the disease.

The true origin of both diseases is the chemical, rather than the mechanical action of the primary infective agent. The infective agent in diphtheria is the bacillus, generally called the Klebs-Löffler bacillus, from the names of Dr. Klebs, who discovered it, and Dr. Löffler, who first succeeded in isolating it. The action of the bacillus cannot always be traced in the human subject, who often comes under observation only when the disease is more or less advanced. Therefore the course and characteristics of the diphtheritic action may more clearly be seen by experiments on animals. Dr. Sidney Martin injected the poison—a deutero-albumose, accompanied by an organic acid—obtained from diphtheritic tissues, into several rabbits. The result was fever, high at first, but gradually becoming less, until on the fifth day the temperature was normal. Weakness of the hind legs appeared on the first day, and gradually increased till almost complete paralysis arrived. There was also considerable wasting of the body; in one animal where a large dose had been injected, this amounted to half the weight of the body. It was shown, too, that the poison acted most powerfully when small but repeated doses of the poison were administered than when only one large dose was given. This is, of course, the way in which the disease is most frequently caught by human subjects. They are in an atmosphere where the diphtheria bacillus is found; they inhale it, swallow it, take it into their organism in one way or another, not in a single large dose, but in repeated small ones; thus the disease gets hold of them gradually and surely. The bacillus once within the body liberates, as Roux and Gersin inferred from their experiments, a ferment which when absorbed digests the proteids of the body, and forms the albumoses and organic acid we have spoken of. These in turn act on the nerves, which on post mortem examination of the bodies of persons who have died of diphtheria, are usually wasted and disintegrated. Hence arise the paralysis which supervenes in severe cases of diphtheria, and the depression which occurs in all. It seems to be proved that the infective agent in diphtheria, though capable of producing fever, is primarily a nerve-poison. It may, too, be taken as proved that the existence of the membrane over the larynx or pharynx is no more than a symptom of the disease. In some cases only a comparatively small proportion of the diphtheria products can be obtained from the membrane. The chief home of these products is the spleen, and in the increase of our knowledge, still vague and imperfect, of the functions of that organ, will be found the best means of dealing with a disease which at present is marked by so high a mortality.

In infective endocarditis, as in diphtheria and also in anthorax, the spleen is the repository, and probably the manufactory of the chemical poison. The poison in this case, however, produces fever and not paralysis. But both find in the proteids of the spleen the food they require; and in both fibrin is the medium, the soil, in which the micro-organism thrives. In diphtheria the characteristic membrane is composed of fibrin, and in infective endocarditis the fibrin capping the vegetation forms its nidus. But a difference arises in the action of the bacillus. In diphtheria, the bacillus works not so much by itself as by its products, so that while the bacillus itself is found only in the superficial parts of the membrane, the ferment it produces is diffused throughout the body. In infective endocarditis and anthrax it is the micro-organism itself that is diffused through the body; it causes the disease itself, without developing any secondary infective agent. Dr. Martin surmises that tetanus, concerning whose pathology much less is known, may resemble diphtheria in its bacillus excreting a ferment which acts as the principal agent in the causation of the disease, for the bacillus itself is limited to the site of the inoculation.

It is not to be supposed that these poisons stand alone, unrelated to anything else in nature. On the contrary, both the vegetable and animal worlds can show poisons very similar in their action. Dr. Martin has noted in the jequirity seed, poisonous proteids will produce a disease characterised by ecchymoses and sanguineous diarrhœa. Snake venom also produces the same symptoms. Dr. Martin thinks that the jequirity poison resembles that of diphtheria very closely, though as yet he does not know if it also produces nerve degeneration.

In his last paragraph Dr. Martin touches upon the possibility of conferring immunity from any disease by injection of its chemical products. Visionary pathologists have dreamed of finding a "vaccine" as well as a "toxine" in these poisons, but the "vaccine" has never yet been isolated. As these lines are being written comes the news of an "anti-diphtherine," discovered by Dr. Klebs, the discoverer of the diphtheria ba[illegible]illus. After the failure of Dr. Koch's consumption cure, the public at large will hesitate to believe in these inoculative specifics; but none the less will scientists go on trying to discover them. Whether or not they succeed in these efforts, it is certain that on their way they will gain much valuable knowledge as to the primary cause of diseases, which by showing how they can be prevented will confer a more valuable and wide-spread immunity than any inoculation by any specific. The great temple of modern chemistry, with all its marvels, was first begun by searchers for the philosopher's store; and in that very science of chemistry it is no uncommon thing to find the "by-products" of any substance more valuable to the world than itself. Similarly, the by-products of pathological investigation may prove to be the most beneficial to mankind; and as these investigations are at the outset uninteresting to the public and unremunerative to the worker, it is eminently desirable that some such institution as the Local Government Board should provide the means for these studies. Their immediate good is not always apparent to outsiders, but the importance of their ultimate results can hardly be gainsaid.

